# Neurodevelopmental Disorders (NDD) Caused by Genomic Alterations of the Ubiquitin-Proteasome System (UPS): the Possible Contribution of Immune Dysregulation to Disease Pathogenesis

**DOI:** 10.3389/fnmol.2021.733012

**Published:** 2021-09-08

**Authors:** Frédéric Ebstein, Sébastien Küry, Jonas Johannes Papendorf, Elke Krüger

**Affiliations:** ^1^ Institute of Medical Biochemistry and Molecular Biology, University Medicine Greifswald, Greifswald, Germany; ^2^CHU Nantes, Service de Génétique Médicale, Nantes, France; ^3^l’Institut du Thorax, CNRS, INSERM, CHU Nantes, Université de Nantes, Nantes, France

**Keywords:** ubiquitin, proteasome, autoinflammation, neurodevelopmental disorders, protein aggregation

## Abstract

Over thirty years have passed since the first description of ubiquitin-positive structures in the brain of patients suffering from Alzheimer’s disease. Meanwhile, the intracellular accumulation of ubiquitin-modified insoluble protein aggregates has become an indisputable hallmark of neurodegeneration. However, the role of ubiquitin and a fortiori the ubiquitin-proteasome system (UPS) in the pathogenesis of neurodevelopmental disorders (NDD) is much less described. In this article, we review all reported monogenic forms of NDD caused by lesions in genes coding for any component of the UPS including ubiquitin-activating (E1), -conjugating (E2) enzymes, ubiquitin ligases (E3), ubiquitin hydrolases, and ubiquitin-like modifiers as well as proteasome subunits. Strikingly, our analysis revealed that a vast majority of these proteins have a described function in the negative regulation of the innate immune response. In this work, we hypothesize a possible involvement of autoinflammation in NDD pathogenesis. Herein, we discuss the parallels between immune dysregulation and neurodevelopment with the aim at improving our understanding the biology of NDD and providing knowledge required for the design of novel therapeutic strategies.

## Introduction

Neurodevelopmental disorders (NDD) are a broad spectrum of early onset syndromes affecting the development of the central nervous system (CNS) with a prevalence in children that exceeds 15% worldwide (Romero-Ayuso, 2021). Formerly referred to as “mental retardation” NDD are typically characterized by deficits in cognitive function and adaptive behavior (Micai et al., 2020; Hanly et al., 2021). They traditionally encompass a wide range of different neurologic diseases ranging from mild to severe that include intellectual disability (ID), developmental delay (DD), autism spectrum disorder (ASD), cerebral palsy (CP), attention deficit/hyperactivity disorder (ADHD), Down syndrome (DS), bipolar disorders (BP), and epilepsy and schizophrenia (Ismail and Shapiro, 2019). One usually discriminates between NDD and neurodegenerative diseases (ND), the latter being a heterogeneous group of late-onset disorders marked by the intracellular accumulation of insoluble protein aggregates perturbing CNS function (Johnson, 2000; Koziorowski et al., 2021). Prominent ND include Alzheimer’s disease (AD), Parkinson’s disease (PD), Huntington’s disease (HD), amyotrophic lateral sclerosis (ALS), and Lewy body dementia (LBD) which all mostly affect elderly individuals (Popa-Wagner et al., 2020; Tittelmeier et al., 2020). The NDD/ND dichotomy is, however, not strict since neurodegeneration may in some cases accompany neurodevelopmental anomalies and vice versa (Thibaut, 2018).

Brain pathologies such as NDD and/or ND are complex disorders which are caused for the most part by genetic and/or environmental factors (Cardoso et al., 2019; Dunn et al., 2019). For instance, unquestionable risk factors for the development of NDD include prenatal asphyxia (Adhikari and Rao, 2017) as well as exposure to ethanol (Sokol et al., 2003), heavy metals (Ijomone et al., 2020), and/or organic pollutants (Mesnil et al., 2020). The genetic components of a large fraction of these psychiatric disorders are difficult to unravel since most of them are not necessarily Mendelian (dominant, recessive, or X-linked) and involve the participation of allelic variants in several genes (Au et al., 2020; Savatt and Myers, 2021). However, it is estimated that approximately 40% of NDD are monogenic conditions predominantly due to lesions of a single gene (Deciphering Developmental, and Disorders, 2017; Brunet et al., 2021), and this figure even rises to about 50% in the case of ID (Kaufman et al., 2010; Karam et al., 2015; Reichenberg et al., 2016; Vissers et al., 2016). Because many of these vulnerable genes do not necessarily encode proteins specifically expressed in the brain with documented functions in neurodevelopment, our current understanding of disease pathogenesis remains extremely limited.

Hence, since their initial descriptions, increasing efforts have been made to better understand how NDD/ND emerge from deteriorated genes. One major breakthrough in this field was made by identification of ubiquitin-positive inclusion bodies in the brain of patients with AD (Mori et al., 1987), which led to the assumption that dysfunctions of the ubiquitin-proteasome system (UPS) may contribute to neurodegeneration. This notion was confirmed 1 year later by a work from the same group showing that Lewy bodies in the brain of six cases with LBD and PD were enriched with ubiquitin (Kuzuhara et al., 1988). Shortly afterward, it became evident that the accumulation of ubiquitin aggregates was not necessarily a histological hallmark restricted to neurodegeneration, but could also be found in the brain of children suffering from various NDD (Del Bigio et al., 1997). Meanwhile, the constantly increasing number of genomic alterations in genes encoding components of the UPS identified in patients with neurological phenotypes unambiguously points to its participation in the pathogenesis of psychiatric disorders. Nevertheless, the extreme versatility of the UPS makes it difficult to fully pinpoint its precise implication in disease pathogenesis, as discussed below.

## The Ubiquitin-Proteasome System (UPS)

The UPS is a highly conserved pathway across eukaryotic species which ensures the rapid elimination of ubiquitin-tagged proteins by the 26S proteasome (Cetin et al., 2021). The ability of the UPS to remove virtually any type of protein substrate makes it indispensable for almost –if not all– basic cellular processes such as cell division, gene expression and signal transduction (Ebstein et al., 2012). A prerequisite for protein breakdown by 26S proteasomes is the covalent modification of intracellular targets with ubiquitin molecules (Wilkinson et al., 1980; Pickart, 2001, 2004; Pickart and Eddins, 2004). In this process, also referred to as ubiquitination (or ubiquitylation), three enzymes (i.e., E1, E2, and E3) catalyze the coordinated transfer of ubiquitin moieties to acceptor residues of proteins destined for degradation (Haas and Siepmann, 1997). Ubiquitination requires the activation of ubiquitin by a E1 ubiquitin-activating enzyme in an ATP-dependent reaction prior to its subsequent transfer onto a E2 ubiquitin-conjugating enzyme. With the help of E3 ubiquitin ligases, the charged E2-ubiquitin transfer ensures the ubiquitination of protein substrates on lysine, cysteine, serine or threonine residues (Tait et al., 2007; McDowell et al., 2010; Golnik et al., 2016; Swatek and Komander, 2016). Depending on their mode of ubiquitin transfer, E3 ubiquitin ligases can be divided into RING-, HECT- and RBR-type E3 ubiquitin ligases (Petroski and Deshaies, 2005; Metzger et al., 2012; Metzger et al., 2014). In contrast to ligases containing the RING (Really Interesting New Gene) finger domain which catalyze the ubiquitin transfer directly from the E2 to substrate proteins, HECT (homologous to E6AP C-terminus)-type E3 ligases first receive ubiquitin from the E2 on a cysteine residue and then transfer it to substrate proteins (Metzger et al., 2012). Among the RING-type ligases, Cullin-RING-type ligases (CRL) are multi-subunit ligases whose major component is a specific cullin (CUL) molecule which itself binds simultaneously to a RING-box protein (Rbx1 or Rbx2) and a substrate receptor (via an adaptor subunit in some cases) at its C- and N-terminus, respectively, (Harper and Schulman, 2021). Because RING-box proteins recruit conjugated E2, CUL are widely regarded as scaffold molecules bridging E2 to substrate proteins. Typical substrate receptors include F-BOX proteins, BTB domain-containing proteins and DCAF proteins which are ligands for CUL1/7, CUL3, and CUL4, respectively, (Harper and Schulman, 2021). Finally, the RBR (RING-in -between RING)-type E3 ubiquitin ligase family includes members that transfer ubiquitin to substrates in a non-canonical manner via a RING/HECT combined process (Uchida and Kitagawa, 2016).

The tagging of intracellular proteins with one ubiquitin moiety is referred to as mono-ubiquitination and is widely viewed as a post-translational process regulating subcellular localization (Sigismund et al., 2004) and gene expression (Marsh et al., 2020). Multiple mono-ubiquitination, namely the addition of one ubiquitin molecule on multiple sites of the same substrate occurs as well and signals either endocytosis, protein trafficking and lysosomal degradation or proteasome-mediated degradation (Livneh et al., 2017).

Most importantly, the ubiquitin molecule itself may be subjected to ubiquitin modification on either one of its eight acceptor sites (K6, K11, K27, K29, K33, K48, K63, and Met-1), thereby generating poly ubiquitin chains carrying distinct ubiquitin linkages. The linkage type determines both the topology of the poly ubiquitin chain and the outcome of the modified substrate. It is well established that poly ubiquitin chains bearing K48-linkages typically deliver the modified protein for subsequent degradation by 26S proteasomes (Pickart and Fushman, 2004). The 26S proteasome is a multi-subunit complex consisting of a 19S regulatory particle and a barrel-shaped 20S core particle (Dahlmann, 2005; Tanaka et al., 2012). While the 19S regulatory particle is specialized in ubiquitin recognition and removing as well as substrate unfolding, the 20S core particle ensures protein breakdown into short peptides via its catalytic β-subunits (Finley et al., 2016; Bard et al., 2018). Substrates modified with K48-linked are rapidly recognized by the ubiquitin receptors PSMD4 and ADRM1 on the 19S regulatory particle which facilitate their translocation into the 20S core particle (Deveraux et al., 1995; Husnjak et al., 2008). The binding of ubiquitin-modified proteins with 26S proteasomes is usually strengthened with the help of protein shuttles which are capable of interacting with both ubiquitin chains and proteasomes via their UBA and UBL domains, respectively, (Chen et al., 2016).

The degradation signal exemplified by K48-linked ubiquitin chains represents just the tip of the iceberg of the ubiquitin code, as the other seven ubiquitination sites of ubiquitin may be used either singly or in combination to generate homotypic or mixed poly ubiquitin chains, respectively, that convey multiple cellular functions including lysosomal targeting and DNA repair to name a few (Akutsu et al., 2016; Grumati and Dikic, 2018). Complexity to the UPS pathway arises further with the existence of ubiquitin-like modifiers which, via a conjugation process similar to that of ubiquitin, are capable of modifying cellular targets in a covalent manner. Ubiquitin-like proteins encompass the ISG15, FAT10, NEDD8, URFM1, UFM1, and ATG12 modifiers as well as those of the ATG8 and SUMO families (Cappadocia and Lima, 2018). Thanks to their ability to tag intracellular substrates, ubiquitin-like modifiers generate an extreme variety of signals including proteolytic and non-proteolytic ones (Streich Jr., and Lima, 2014). Strikingly, SUMO, and to a lesser extent NEDD8 and ISG15, may themselves be subjected to ubiquitination at various lysine residues, thereby giving rise to hybrid chains whose biological functions, however, have not been fully elucidated (Perez Berrocal et al., 2019; Mulder et al., 2020).

Importantly, both ubiquitin and ubiquitin-like modifications are reversible processes which can be counteracted anytime by ubiquitin hydrolases (also called deubiquitinating enzymes, DUB). Up to now, an approximate number of 100 DUB have been identified, whereby the largest families are represented by the ubiquitin-specific proteases (USP), the ovarian tumor proteases (OTU), and ubiquitin C-terminal hydrolases (UCH) (Clague et al., 2019). Many DUB and E3 ubiquitin ligases regulate fundamental cellular pathways including cell division or death, genomic integrity, epigenetic control, developmental, and differentiation pathways as well as cellular homeostasis (Morgan and Crawford, 2021).

**FIGURE 1:**
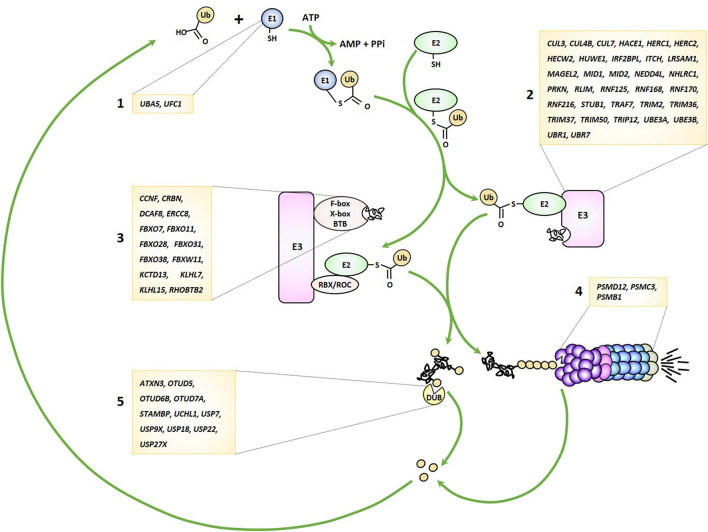
Genetic lesions identified in patients with NDD may affect any stage of the UPS pathway. UPS-related genes disrupted in NDD encode various components of the ubiquitin-conjugation system including E1 ubiquitin-activating enzymes (1), E3 ubiquitin ligases (2), Cullin-RING-type E3 ubiquitin ligases (3), proteasome subunits (4) and deubiquitinating enzymes (5), as indicated.

As alluded to earlier, the UPS pathway is frequently damaged in several forms of NDD by genomic alterations that may affect either one of the many genes encoding its various components. Because virtually any gene seems vulnerable, any stage of this process may be impaired from ubiquitin transfer to ubiquitin removal and/or proteasome-mediated breakdown of ubiquitin-modified proteins (Figure 1). These observations clearly point to a cause-and-effect relationship between perturbed UPS function and NDD onset, as discussed below.

## E3 Ubiquitin Ligases in NDD

Ubiquitin ligases are by far the largest group of UPS genes identified as NDD causing-genes. The first identified member of this constantly growing family is the *UBE3A* gene encoding the E6-AP HECT-type E3 ubiquitin ligase and whose loss-of-function has been shown to cause Angelman syndrome more than twenty years ago (Kishino et al., 1997; Matsuura et al., 1997; Sutcliffe et al., 1997). Because *UBE3A* is exclusively expressed from the maternal allele in neurons, any deletion or point mutations affecting the maternal chromosome leads to loss of E6-AP expression in these cells and result in the acquisition of a neuronal phenotype mostly characterized by absent speech, intellectual disability and happy demeanor with unusually frequent laughing smiling (Maranga et al., 2020).

Since the original reports associating *UBE3A* with Angelman syndrome in 1997, approximately forty-five further genes coding for E3 ubiquitin ligases or CRL substrate receptors have been identified as causative genes for forty-eight different forms of NDD (Tables 1, 2). Clinical features commonly observed in all these syndromes include developmental delay, cognitive deficits, dysmorphic facial features, hypotonia and seizures. However, given the large variety of signals generated by E3 ubiquitin ligases, the phenotypic spectrum of NDD subjects with loss-of-function in E3 genes may vary to a large degree. Herein, limb anomalies such as brachydactyly, polydactyly, or camptodactyly are frequently detected in patients carrying genomic alterations in the *HUWE1*, *TRAF7*, *UBE3B*, *ITCH*, or *FBXW11* genes (Buntinx and Majewski, 1990; Lohr et al., 2010; Moortgat et al., 2018; Tokita et al., 2018; Holt et al., 2019), while gonadal dysfunction seems to be restricted to a subset of NDD cases carrying variants of the *RNF216*, *STUB1*, *TRIM37*, or *KLHL15* genes (Seminara et al., 2002; Jagiello et al., 2003; Heimdal et al., 2014; Mignon-Ravix et al., 2014).

**TABLE 1:** NDD-causing genes encoding E3 ubiquitin ligases and associated syndromes.

Gene	OMIM	Syndrome	References	Described regulator of:	References
*CUL3*	619239	NEURODEVELOPMENTAL DISORDER WITH OR WITHOUT AUTISM OR SEIZURES	Nakashima et al. (2020)	T-cell function	Mathew et al. (2012)
*CUL4B*	300354	MENTAL RETARDATION, X-LINKED, SYNDROMIC, CABEZAS TYPE	Tarpey et al. (2007)	NF-κB signaling	Hung et al. (2014); Song et al. (2021)
*CUL7*	273750	THREE M SYNDROME 1	Huber et al. (2005)	Ig class switch recombination	Luo et al. (2019)
*HACE1*	616756	SPASTIC PARAPLEGIA AND PSYCHOMOTOR RETARDATION WITH OR WITHOUT SEIZURES	Hollstein et al. (2015)	Antiviral immunity	Mao et al. (2016)
*HERC1*	617011	MACROCEPHALY, DYSMORPHIC FACIES, AND PSYCHOMOTOR RETARDATION	Nguyen et al. (2016)	MAP kinase and mTOR signaling	Sala-Gaston et al. (2020)
*HERC2*	615516	MENTAL RETARDATION, AUTOSOMAL RECESSIVE 38	Morice-Picard et al. (2016)	Genomic stability	Sala-Gaston et al. (2020)
*HECW2*	617268	NEURODEVELOPMENTAL DISORDER WITH HYPOTONIA, SEIZURES, AND ABSENT LANGUAGE	Berko et al. (2017)	Mitotic metaphase/anaphase transition, heterochromatin packaging	Lu et al. (2013); Krishnamoorthy et al. (2018)
*HUWE1*	309590	MENTAL RETARDATION, X-LINKED, SYNDROMIC, TURNER TYPE	Froyen et al. (2008)	Inflammasome, NF-κB signaling	Guo et al. (2020b); Ohtake et al. (2016)
*IRF2BPL*	618088	NEURODEVELOPMENTAL DISORDER WITH REGRESSION, ABNORMAL MOVEMENTS, LOSS OF SPEECH, AND SEIZURES	Tran Mau-Them et al. (2019)	Apoptosis, survival, and cell differentiation	Ramalho-Oliveira et al. (2019)
*ITCH*	613385	AUTOIMMUNE DISEASE, MULTISYSTEM, WITH FACIAL DYSMORPHISM	Lohr et al. (2010)	Inflammation, T-cell differentiation	Field et al. (2020)
*LRSAM1*	614436	CHARCOT-MARIE-TOOTH DISEASE, AXONAL, TYPE 2P	Guernsey et al. (2010)	Antibacterial autophagic response	Ng et al. (2011)
*MAGEL2*	615547	SCHAAF-YANG SYNDROME	Ates et al. (2019)	Immune infiltration	Arora et al. (2020)
*MID1*	300000	OPITZ GBBB SYNDROME	Quaderi et al. (1997)	T-cell differentiation, Antiviral immunity	Collison et al. (2013); Chen et al. (2021)
*MID2*	300928	MENTAL RETARDATION, X-LINKED 101	Geetha et al. (2014)	Cytokinesis	Zanchetta and Meroni (2019)
*NEDD4L*	617201	PERIVENTRICULAR NODULAR HETEROTOPIA 7	Broix et al. (2016)	Antiviral immunity	Gao et al. (2021)
*NHLRC1*	254780	EPILEPSY, PROGRESSIVE MYOCLONIC, 2B, INCLUDED	Chan et al. (2003)	Inflammatory cytokines	Lopez-Gonzalez et al. (2017)
*PRKN*	600116	PARKINSON DISEASE 2, AUTOSOMAL RECESSIVE JUVENILE	Kitada et al. (1998)	Antiviral immunity	Sliter et al. (2018)
*RLIM*	300978	TONNE-KALSCHEUER SYNDROME	Tonne et al. (2015)	Imprinted X chromosome inactivation	Gontan et al. (2018)
*RNF125*	616260	TENORIO SYNDROME	Tenorio et al. (2014)	Antiviral immunity, inflammasome IL-36 signaling	Oshiumi et al. (2010); Jia et al. (2017); Saha et al. (2018); Tang et al. (2020)
*RNF168*	611943	RIDDLE SYNDROME	Stewart et al. (2009)	Ig class switch recombination, Immune deficiency	Ramachandran et al. (2010); Chinn et al. (2017)
*RNF170*	608984	ATAXIA, SENSORY, 1, AUTOSOMAL DOMINANT	Valdmanis et al. (2011)	Antiviral immunity	Song et al. (2020)
*RNF216*	212840	GORDON HOLMES SYNDROME	Margolin et al. (2013)	TLR signaling, Antiviral immunity	Nakhaei et al. (2009); Kumazoe et al. (2017)
*STUB1*	615768; 618093	SPINOCEREBELLAR ATAXIA, AUTOSOMAL RECESSIVE 16; SPINOCEREBELLAR ATAXIA 48	Shi et al. (2013); Genis et al. (2018)	TLR signaling, T-cell function, antiviral immunity, IL-4 signaling	Yang et al. (2011); Chen et al. (2013); Wei et al. (2014); Zhao et al. (2016); Zhou et al. (2018)
*TRAF7*	618164	CARDIAC, FACIAL, AND DIGITAL ANOMALIES WITH DEVELOPMENTAL DELAY	Tokita et al. (2018)	NF-κB signaling	Zotti et al. (2011)
*TRIM2*	615490	CHARCOT-MARIE-TOOTH DISEASE, AXONAL, TYPE 2R	Ylikallio et al. (2013)	New World arenavirus entry	Sarute et al. (2019)
*TRIM36*	206500	ANENCEPHALY	Singh et al. (2017)	Cell cycle progression	Miyajima et al. (2009)
*TRIM37*	253250	MULIBREY NANISM	Kallijarvi et al. (2002)	NF-κB signaling, Inflammation	Li et al. (2018); Zhao et al. (2021)
*TRIM50*	194050	WILLIAMS-BEUREN SYNDROME	Micale et al. (2008)	Clearance of aggresomes polyubiquitinated	Fusco et al. (2014)
*TRIP12*	617752	CLARK-BARAITSER SYNDROME	Zhang et al. (2017)	Epithelial-mesenchymal transition, DNA repair	Challa et al. (2021); Lee et al. (2021)
*UBE3A*	105830	ANGELMAN SYNDROME	Kishino et al. (1997)	Antiviral immunity	Furumai et al. (2019)
*UBE3B*	244450	KAUFMAN OCULOCEREBROFACIAL SYNDROME	Basel-Vanagaite et al. (2012)	Cell proliferation	Li et al. (2020)
*UBR1*	243800	JOHANSON-BLIZZARD SYNDROME	Zenker et al. (2005)	Protein quality control	Zenker et al. (2005)
*UBR7*	619189	LI-CAMPEAU SYNDROME	Li et al. (2021)	NLR activation, Stem cell function	Zhang et al. (2019); Srivastava et al. (2021)

*The potential implication of the identified gene products in the regulation of innate and/or adaptive is indicated. When available, the OMIM (Online Mendelian Inheritance in Man^®^) disorder number is also reported.*

**TABLE 2:** NDD-causing genes encoding CUL substrate receptors and associated syndromes.

Gene	E3 Ubiquitin ligase	OMIM	Syndrome	References	Described regulator of:	References
*CCNF*	SKP1-CUL1-F-box	619141	FRONTOTEMPORAL DEMENTIA AND/OR AMYOTROPHIC LATERAL SCLEROSIS 5	Williams et al. (2016)	HIV infectivity in CD4 + T-cells	Augustine et al. (2017)
*CRBN*	DDB1-CUL4-X-box	607417	MENTAL RETARDATION, AUTOSOMAL RECESSIVE 2	Higgins et al. (2004)	TLR signaling, T-cell function	Millrine et al. (2016); Min et al. (2016); Yang et al. (2018); Hesterberg et al. (2020)
*DCAF8*	DDB1-CUL4-X-box	610100	GIANT AXONAL NEUROPATHY 2	Klein et al. (2014)	Inflammatory cytokines	Peng et al. (2020)
*ERCC8*	DDB1-CUL4-X-box	216400 614621	COCKAYNE SYNDROME A UV-SENSITIVE SYNDROME 2	Nardo et al. (2009)	Inflammation	Ku and Cheng (2020)
*FBXO7*	SKP1-CUL1-F-box	260300	PARKINSON DISEASE 15, AUTOSOMAL RECESSIVE EARLY-ONSET	Shojaee et al. (2008)	NF-κB signaling	Kuiken et al. (2012)
*FBXO11*	SKP1-CUL1-F-box	618089	INTELLECTUAL DEVELOPMENTAL DISORDER WITH DYSMORPHIC FACIES AND BEHAVIORAL ABNORMALITIES	Gregor et al. (2018)	Inflammation, TGF-β signaling	Hardisty-Hughes et al. (2006); Tateossian et al. (2009)
*FBXO28*	SKP1-CUL1-F-box		DEVELOPMENTAL DELAY, DYSMORPHIC FEATURES, AND INTRACTABLE EPILEPSY	Balak et al. (2018)	Mitochondrial function	Zou et al. (2016)
*FBXO31*	SKP1-CUL1-F-box	615979	MENTAL RETARDATION, AUTOSOMAL RECESSIVE 45	Mir et al. (2014)	Stem cell differentiation	Baek et al. (2021)
*FBXO38*	SKP1-CUL1-F-box	615575	NEURONOPATHY, DISTAL HEREDITARY MOTOR, TYPE IID	Sumner et al. (2013)	T-cell function	Meng et al. (2018)
*FBXW11*	SKP1-CUL1-F-box	618914	NEURODEVELOPMENTAL, JAW, EYE, AND DIGITAL SYNDROME	Holt et al. (2019)	Ig class switch recombination	Luo et al. (2019)
*KCTD13*	BTB-CUL3-RBX1	611913; 614671	CHROMOSOME 16p11.2 DELETION SYNDROME; CHROMOSOME 16p11.2 DUPLICATION SYNDROME	Crepel et al. (2011)	Cell motility	Chen et al. (2009)
*KLHL7*	BTB-CUL3-RBX1	617055	PERCHING SYNDROME	Friedman et al. (2009)	Nucleolar integrity	Kim et al. (2017)
*KLHL15*	BTB-CUL3-RBX1	300982	MENTAL RETARDATION, X-LINKED 103	Mignon-Ravix et al. (2014)	DNA end resection	Ferretti et al. (2016)
*RHOBTB2*	BTB-CUL3-RBX1	618004	DEVELOPMENTAL AND EPILEPTIC ENCEPHALOPATHY 64	Straub et al. (2018)	Vesicle trafficking	Ji and Rivero (2016)

*The potential implication of the gene products in the regulation of innate and/or adaptive is indicated. When available, the OMIM (Online Mendelian Inheritance in Man^®^) disorder number is also reported.*

## E3 Ubiquitin Ligases and Protein Aggregation in NDD

Over the last two decades, many attempts have been made to unravel the molecular pathogenesis of NDD caused by ubiquitin ligase dysfunction. One straightforward route to address this point consists of identifying downstream target substrates of the affected ligases by proteomic-based methods (Rayner et al., 2019). This strategy is nevertheless hampered by the fact that E3 ubiquitin ligases may have multiple substrates which themselves may fulfill many different functions. One prime example of such ligases is CHIP encoded by the *STUB1* gene and whose genomic alterations cause spinocerebellar ataxia (Shi et al., 2013). Thanks to its ability to bind to cellular chaperones such as HSP70 and HSP90, CHIP mediates the ubiquitination of misfolded proteins, thereby targeting them for subsequent degradation by 26S proteasomes (Edkins, 2015; Wang et al., 2020). Such misfolded proteins typically encompass defective ribosomal products (DRIPS) which are generated during protein biosynthesis as a consequence of ribosomal mistranslation (Yewdell et al., 1996). Pioneering work of J. Yewdell and colleagues has estimated that DRIPS may account for 25% of the total pool of newly synthetized proteins in eukaryotic cells (Schubert et al., 2000; Princiotta et al., 2003; Qian et al., 2006). This implies that virtually any intracellular protein may become a target of CHIP, making it impossible to associate CHIP defects with one particular cellular pathway and/or function. The extremely broad substrate specificity of CHIP also presupposes that *STUB1* loss-of-function results in the accumulation of various misfolded and/or damaged proteins that fail to undergo ubiquitination. Whether these protein aggregates are toxic as a whole and contribute to the pathogenesis of spinocerebellar ataxia is unclear. A fortiori, these inclusions would be devoid of ubiquitin molecules and, as such, not reminiscent of those typically accumulating during neurodegeneration. Herein, this assumption would underline a major distinction between NDD and ND, as it would preclude that the perturbations of protein homeostasis associated with NDD are not due to proteolytic dysfunction. Besides DRIPS, one cannot exclude that NDD due to *STUB1* loss-of-function mutations may occur as a consequence of the inability of the cells to remove specific full-length mature proteins, which would then drive the disease by perturbing specific cellular pathways.

Like CHIP, UBR1, and UBR7, whose deficiencies reportedly cause the Johanson-Blizzard (Zenker et al., 2005) and Li-Campeau syndromes (Li et al., 2021), respectively, are E3 ubiquitin ligases with multiple potential substrates. As members of the N-end rule pathway, UBR1 and UBR7 target any protein carrying N-terminal destabilizing motifs (also referred to as “N-degrons”) such as arginine residues for degradation (Varshavsky, 2019). Substrates of the N-end rule pathway physiologically arise from limited proteolysis and encompass a wide variety of intracellular proteins fulfilling various functions in cell signaling, cellular homeostasis and apoptosis (Varshavsky, 2019). Herein, the multitude of pathways potentially affected by UBR1 and/or UBR7 loss-of-function mutations substantially challenges our understanding of NDD pathophysiology. In addition, one cannot exclude that the diseases may be triggered by the unspecific accumulation of N-end rule substrates that would affect cell function and/or integrity. In any case, CHIP, UBR1 and UBR7 exemplify the difficulty of deciphering the molecular pathogenesis of syndromes due to E3 ubiquitin ligase which have multiple substrates.

## NDD-Associated E3 Ubiquitin Ligases and Their Roles in the Immune Response

Another E3 ubiquitin ligase potentially causing NDD with a wide range of substrates is ITCH, whose genetic disruption has been shown to cause a syndromic multisystem autoimmune disease referred to as autoimmune disease, multisystem, with facial dysmorphism (ADMFD) (Lohr et al., 2010). Interestingly, ADMFD is also a neurological disease with affected children exhibiting typical NDD features while developing autoimmune systemic responses at the same time. The immunological component of ADMFD is not surprising in view of the substantial number of ITCH cellular targets which play critical roles in T- and B-cell function. These notably include the T-cell receptor (TCR) chain-ζ as well as the RAR-related orphan receptor (ROR)-γt transcription factor, which control T-cell signaling and differentiation, respectively (Huang et al., 2010; Kathania et al., 2016).

**FIGURE 2:**
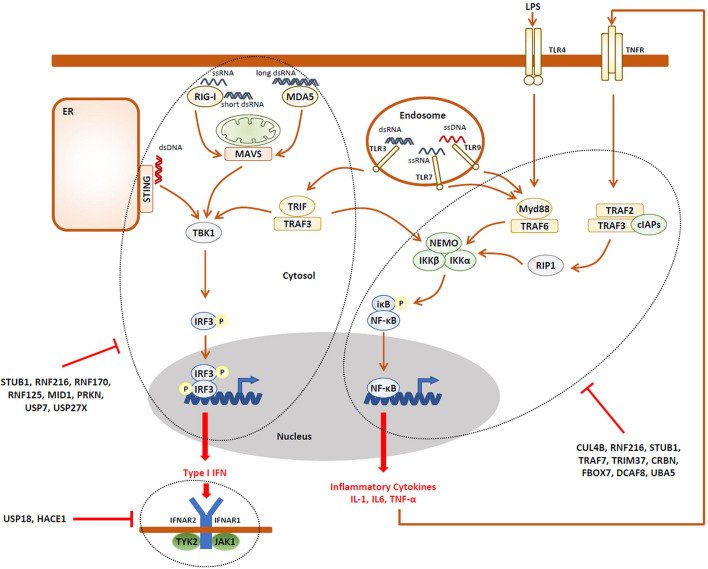
A large fraction of the UPS gene products associated with NDD are negative regulators of the innate immune response. Activation of pathogen recognition receptors (PRR, i.e., TLR3, TLR4, TLR7, TLR9, RIG-1, MDA5, and STING) by pathogen-associated molecular patterns (PAMP, i.e., LPS, ssRNA, dsRNA, and CpG) results in the activation of intracellular signaling cascades which ultimately promote the nuclear translocation of the IRF3 and NF-κB transcriptions factors, as indicated. These, in turn, directly induce the expression of pro-inflammatory cytokines (i.e., TNF-α) and type I IFN which engage the innate arm of the immune system through autocrine and/or paracrine loops. Both the IRF3 and NF-κB activation pathways are down-regulated by many components of the UPS which have been associated with NDD, as indicated. In addition, the autocrine/paracrine action of type I IFN is subjected to negative regulation by the UPS genes USP18 and HACE1, as indicated.

The observation that NDD may be accompanied by immune manifestations is somehow intriguing and raises the question as to whether an unrestrained innate and/or adaptive immune response (i.e., autoinflammation and/or autoimmunity) might underlie the pathogenesis of NDD. Strikingly, besides ITCH, more than two-thirds of the E3 ubiquitin ligases reported to cause NDD have critical functions in the innate and adaptive immune systems. As listed in Tables 1, 2, the E3 ubiquitin ligases CUL4B (Hung et al., 2014; Song et al., 2021), HUWE1 (Ohtake et al., 2016; Guo et al., 2020b), RNF216 (Kumazoe et al., 2017), STUB1 (Yang et al., 2011), TRAF7 (Zotti et al., 2011), TRIM37 (Li et al., 2018; Zhao et al., 2021), CRBN (Min et al., 2016; Yang et al., 2018), and the substrate recognition component FBXO7 (Kuiken et al., 2012) have been shown to regulate the expression of inflammatory cytokines mostly thanks to their capacity of modulating NF-κB signaling and/or the inflammasome. It is worth noting that, except HUWE1, all these ligases are described as inflammation negative regulators of these pathways (Figure 2), implying that any loss-of-function of any one of these genes would result in the sustained production of pro-inflammatory cytokines.

It is also understood that the E3 ubiquitin ligases HACE1 (Mao et al., 2016), MID1 (Chen et al., 2021), NEDD4L (Gao et al., 2021), PRKN (Sliter et al., 2018), RNF125 (Arimoto et al., 2007), RNF170 (Song et al., 2020), RNF216 (Nakhaei et al., 2009), STUB1 (Zhou et al., 2018), as well as UBE3A (Furumai et al., 2019) are involved in antiviral innate defense and the generation of type I interferon (IFN) responses (Figure 2). Again, besides NEDD4L, all these genes encode ligases involved in type I IFN negative feedback loops and, as such any dysfunction, would lead to uncontrolled type I IFN responses.

Some other NDD ubiquitin and/or CRL ligases seem to exert their activity predominantly during the adaptive immune response. These include CUL7 together with the substrate receptor FBXW11 (Luo et al., 2019) as well as RNF168 (Ramachandran et al., 2010) which regulate immunoglobulin switch recombination or CUL3 (Mathew et al., 2012), ITCH (Field et al., 2020), MID1 (Collison et al., 2013), STUB1 (Chen et al., 2013), CRBN (Hesterberg et al., 2020), and FBXO38 (Meng et al., 2018) that are involved in T-cell function and/or differentiation.

Despite the prominent implication of ligases in multiples levels of the innate immune system, only a very small number of NDD cases are associated with symptoms of autoinflammation. These include the Tenorio syndrome caused by RNF125 deficiency which, beside syndromic intellectual disability, leads to a severe inflammatory phenotype with recurrent episodes of conjunctivitis and stomatitis (Tenorio et al., 2014). The immune manifestations of the Tenorio syndrome clearly corroborate the fact that RNF125 acts as a negative regulator of innate immune signaling by targeting key pattern recognition receptors (i.e., RIG-1, MDA5) for degradation (Arimoto et al., 2007, 2018). Also found in this category of NDD are the Williams-Beuren and Cockayne syndrome which are characterized by intracranial calcifications (Neill and Dingwall, 1950; Knudtzon et al., 1987; Wilson et al., 2016), a typical trait of neuroinflammation (Saade et al., 2019). Ironically, and in contrast to the Tenorio syndrome, both Williams-Beuren and Cockayne syndromes are caused by genomic alterations in genes encoding E3 ubiquitin ligases (i.e., *TRIM50* and *ERCC8*) with no described function in the immune system. Non-etheless, a closer look at their cellular targets reveals that both of these ligases may intersect with host innate immune defenses. In effect, one major substrate of TRIM50 includes BECN1 (Fusco et al., 2018), an important component of the autophagy lysosomal degradation pathway that recruits autophagy proteins to the phagophore assembly site (Kang et al., 2011). Interestingly, it has been shown that BECN1 is capable of activating NF-κB (Leonard et al., 2019), suggesting that any perturbations of its turnover due to TRIM50 deficiency might result in sustained inflammatory responses. In a similar manner, because of its implication in transcription-coupled nucleotide excision repair (TC-NER) in response to ultraviolet (UV) irradiation (Nardo et al., 2009), ERCC8 may render the cells susceptible for autoinflammation. In fact, it is conceivable that ERCC8 loss-of-function might result in abnormal cytosolic accumulation of damaged transcripts, which in turn may be sensed as non-self RNA by immune cells.

The lack of peripheral immune manifestations in NDD caused by the disruption of other E3 ubiquitin ligases may seem surprising at first sight, but it does not necessarily preclude the absence of ongoing autoinflammation and/or autoimmunity in these patients. Indeed, immune-inflammatory parameters have been frequently detected in NDD subjects seemingly devoid of clinical inflammatory symptoms. For instance, an elevated pro-inflammatory cytokine blood profile has been reported in patients with ASD (Eftekharian et al., 2018; Matta et al., 2019), epilepsy (Riazi et al., 2010), schizophrenia (Potvin et al., 2008; Miller et al., 2011; Tourjman et al., 2013), BP (Benedetti et al., 2020), and ADHD (Zhou et al., 2017). This also holds true for both Aicardi-Goutières and Down syndromes, two NDD which fail to exhibit clinical features of systemic inflammation but fall into the category of interferonopathies because of their sustained production of type I IFN (Crow and Manel, 2015; Livingston and Crow, 2016; Sullivan et al., 2016; Sullivan et al., 2017; Waugh et al., 2019).

### Deubiquitinating Enzymes (DUB) in NDD

As illustrated in Table 3, a total of ten DUB have been reported as diseases-causing genes for various forms of NDD. Strikingly, eight of them have described roles in the immune system. These include STAMBP (Bednash et al., 2017, 2021), UCHL1 (Karim et al., 2013), USP7 (Daubeuf et al., 2009; Colleran et al., 2013; Palazon-Riquelme et al., 2018), and USP9X (Xiang et al., 2019) which have all been shown to modulate the expression of pro-inflammatory cytokines thanks to their capacity of interfering with the NF-κB signaling and/or inflammasome pathways. Other DUB exert a more specific action on type I IFN responses, rendering them essential at the very first line of innate antiviral defense. One prominent member of this family is undoubtedly USP18 which negatively regulates type I IFN signaling by competing with Janus kinase 1 (JAK1) for binding to IFN α/β receptor 2 (IFNAR2) (Malakhova et al., 2006). The observation that USP18 loss-of-function mutations give rise to brain malformations in patients with pseudo-Torch syndrome (Meuwissen et al., 2016) strongly suggests a cause-and-effect relationship between type I IFN and neurodevelopmental disabilities. Further support for this notion comes from the identification of USP7 and USP27X as disease-causing genes for the Hao-Fountain syndrome and X-linked mental retardation, respectively, (Hao et al., 2015; Hu et al., 2016). Both of these genes encode DUB that stimulate a type I IFN negative feedback mechanism by removing K63-linked poly ubiquitin chains on critical components of the antiviral signaling pathway such as RIG-I (Tao et al., 2020) and TBK1 (Cai et al., 2018).

**TABLE 3:** NDD-causing genes encoding DUB and associated syndromes.

Gene	OMIM	Syndrome	References	Described regulator of:	References
*ATXN3*	109150	MACHADO-JOSEPH DISEASE	Kawaguchi et al. (1994)	Antiviral immunity	Feng et al. (2018)
*OTUD5*	301056	MULTIPLE CONGENITAL ANOMALIES-NEURODEVELOPMENTAL SYNDROME, X-LINKED	Beck et al. (2021); Tripolszki et al. (2021)	Antiviral immunity	Guo et al. (2020a)
*OTUD6B*	617452	INTELLECTUAL DEVELOPMENTAL DISORDER WITH DYSMORPHIC FACIES, SEIZURES, AND DISTAL LIMB ANOMALIES	Santiago-Sim et al. (2017)	B-lymphocyte proliferation	Xu et al. (2011)
*OTUD7A*	612001	CHROMOSOME 15q13.3 DELETION SYNDROME	Hoppman-Chaney et al. (2013)	DNA repair	Wu et al. (2019)
*STAMBP*	614261	MICROCEPHALY-CAPILLARY MALFORMATION SYNDROME	Hori et al. (2018)	Inflammasome	Bednash et al. (2017, 2021)
*UCHL1*	615491, 613643	SPASTIC PARAPLEGIA 79, PARKINSON DISEASE 5, AUTOSOMAL DOMINANT	Leroy et al. (1998)	TLR signaling	Karim et al. (2013)
*USP7*	616863	HAO-FOUNTAIN SYNDROME	Hao et al. (2015)	TLR signaling, antiviral immunity, NF-κB signalling, T-cell differentiation, Inflammasome	Daubeuf et al. (2009); Colleran et al. (2013); van Loosdregt et al. (2013); Cai et al. (2018); Palazon-Riquelme et al. (2018)
*USP9X*	300919; 300968	MENTAL RETARDATION, X-LINKED 99; MENTAL RETARDATION, X-LINKED 99, SYNDROMIC, FEMALE-RESTRICTED	Homan et al. (2014)	T-cell signaling; TLR signaling	Naik et al. (2014); Naik and Dixit (2016); Xiang et al. (2019)
*USP18*	617397	PSEUDO-TORCH SYNDROME 2	Meuwissen et al. (2016)	Antiviral immunity	Ritchie et al. (2004)
*USP27X*	300984	MENTAL RETARDATION, X-LINKED 105	Hu et al. (2016)	Antiviral immunity	Guo et al. (2019); Tao et al. (2020)

*The potential implication of the gene products in the regulation of innate and/or adaptive is indicated. When available, the OMIM (Online Mendelian Inheritance in Man^®^) disorder number is also reported.*

The association between type I IFN in NDD pathogenesis is, however, challenged by the fact that two DUB identified as NDD-causing have been also described as potent inducers of type I IFN. These include ATXN3 and OTUD5, respectively, causing the Machado-Joseph disease and a X-linked multiple congenital anomalies-neurodevelopmental syndrome (Kawaguchi et al., 1994; Beck et al., 2021; Tripolszki et al., 2021). Indeed, by removing proteolytic poly ubiquitin chains from the cytosolic DNA sensor STING, OTUD5 facilitates antiviral innate signaling and the subsequent transcription of type I IFN genes (Guo et al., 2020a). Likewise, ATXN3 has been shown to exacerbate type I antiviral response via the deubiquitination and stabilization of histone deacetylase 3 (HDAC3) (Feng et al., 2018). As such, any loss-of-function of any of these DUB would mitigate type I IFN responses and contradicts the view that NDD is associated with increased inflammation. However, it is highly likely that ATX3 deficiency exert its pathogenic effect independent of its deubiquitination function, since Machado-Joseph disease is a triplet (CAG encoding glutamine, Q) repeat expansion disorder whereby ATXN3 mutant proteins accumulate as toxic insoluble protein aggregates (Chai et al., 1999). Quite on the contrary, it has been shown that cell lines expressing expanded ATXN3 were characterized by increased transcription of pro-inflammatory cytokines (Evert et al., 2001; Evert et al., 2006). As for OTUD5, its recently described implication in DNA damage-induced transcriptional repression (de Vivo et al., 2019) opens the possibility that its genomic disruption would generate a danger signal leading to inflammation via excessive cytosolic mRNA accumulation.

Very little is known about the biological function and/or cellular targets of OTUD7A and OTUD6B, whose genetic lesions cause the chromosome 15q13.3 deletion syndrome and an intellectual developmental disorder with dysmorphic facies, seizures, and distal limb anomalies (Santiago-Sim et al., 2017; Uddin et al., 2018), respectively. Interestingly, common to both disorders is a decreased proteasome function ultimately resulting in the aggregation of ubiquitin-modified protein (Santiago-Sim et al., 2017; Garret et al., 2020). These studies suggest that OTUD7A and OTUD6B are directly or indirectly involved in the regulation of proteasome-mediated proteolysis and that their deficiencies would result in cellular situations very similar neurodegeneration ones. A distant and indirect member of the DUB family associated with NDD phenotypes is USP22 which participates in the pathogenesis of spinocerebellar ataxia 7 caused by poly Q repeats in the ATX7 gene (David et al., 1997). Both UPS22 and ATX7 are parts of the Spt-Ada-Gcn5 Acetyl transferase (SAGA) complex which promotes gene transcription via histone acetylation and deubiquitination activities (Melo-Cardenas et al., 2016). It is argued that the generation of poly Q Ataxin-7 protein aggregates substantially affects the access of USP22 to its cellular substrates notably histone H2B (Henry et al., 2003), a dysfunction likely contributing to disease onset. Most interestingly, it has been shown that USP22 gene silencing is accompanied by activation of the JAK-STAT1 signaling pathway (Han et al., 2020), thereby raising the possibility that type I IFN might be a component of spinocerebellar ataxia 7.

### Proteasomes in NDD

As shown in Table 4, the most recent identified group of UPS genes associated with NDD include those encoding proteasome subunits. In this short list are found the *PSMD12*, *PSMC3* and *PSMB1* genes that cause the Stankiewicz-Isidor syndrome (STISS), a neurosensory syndrome combining deafness and cataract as well as a disorder characterized by microcephaly, intellectual disability, developmental delay and short stature, respectively, (Kury et al., 2017; Ansar et al., 2020; Kroll-Hermi et al., 2020). Like most of the NDD due to genomic alterations of UPS genes, these syndromes are seemingly devoid of systemic signs of immune dysregulation. This observation is even more surprising considering the fact that proteasome loss-of-function mutations have been described to cause autoinflammatory diseases referred to as proteasome-associated autoinflammatory syndromes (PRAAS) or chronic atypical neutrophilic dermatosis with lipodystrophy and elevated temperature (CANDLE) (Agarwal et al., 2010; Arima et al., 2011; Kitamura et al., 2011; Liu et al., 2012; Brehm et al., 2015; Poli et al., 2018; de Jesus et al., 2019; Sarrabay et al., 2019; Kataoka et al., 2021). In contrast to the NDD alterations that may affect 20S or 19S proteasome complexes, the CANDLE/PRAAS mutations are exclusively located in the 20S core particle and/or proteasome assembly chaperones (Ebstein et al., 2019). Common to all CANDLE/PRAAS subject is a type I IFN gene signature characterized by increased amounts of transcripts encoding canonical IFN-stimulated genes such as *ISG15*, *SIGLEC-1*, *IFI44L*, *IFIT1*, *IFI27*, and *RSAD2* (Brehm and Krüger, 2015). Interestingly, resetting the immune system of patients with mutation in the proteasome assembly maturation protein (POMP) via hematopoietic stem cell transplantation (HSCT) could successfully reverse the clinical and molecular features of CANDLE/PRAAS (Martinez et al., 2021), indicating that the signature is mostly generated by immune cells. Although some patients may exhibit signs of cognitive impairment, CANDLE/PRAAS are usually not dominated by typical neuropsychological and biological features of a neurodevelopmental disorder, thereby making them distinct from classical NDD. Conversely, and unlike CANDLE/PRAAS, NDD due to lesions in *PSMD12*, *PSMC3* and *PSMB1* genes fail to develop systemic autoinflammation, which prevent them from falling into autoinflammatory disease categories. The reasons why proteasomes loss-of-function mutations lead to two clinically distinct phenotypes are unclear and warrant further investigations. Clearly, the divergence between the two diseases is not dictated by the location of the affected subunit within the 26S proteasome, as initially assumed (Ebstein et al., 2019). The notion that CANDLE/PRAAS develop peripheral autoimmunity is not unexpected given the pleiotropic role of proteasomes in multiple inflammatory signal cascades (Cetin et al., 2021; Goetzke et al., 2021). On the contrary, the lack of systemic manifestations in NDD due to proteasome loss-of-function mutations is intriguing, but again does not necessarily imply the absence of autoinflammation in some tissues and/or the generation of atypical inflammatory signatures that may have been overlooked.

**TABLE 4:** NDD-causing genes encoding proteasome subunits and associated syndromes.

Gene	OMIM	Syndrome	References	Described regulator of	References
*PSMD12*	617516	STANKIEWICZ-ISIDOR SYNDROME	Kury et al. (2017)	Inflammation	Ebstein et al. (2019); Cetin et al. (2021); Goetzke et al. (2021)
*PSMC3*		NEUROSENSORY SYNDROME COMBINING DEAFNESS AND CATARACT	Kroll-Hermi et al. (2020)		
*PSMB1*		MICROCEPHALY, INTELLECTUAL DISABILITY, DEVELOPMENTAL DELAY AND SHORT STATURE	Ansar et al. (2020)		

*The potential implication of the gene products in the regulation of innate and/or adaptive is indicated. When available, the OMIM (Online Mendelian Inheritance in Man^®^) disorder number is also reported.*

### UFMylation in NDD

As shown in Table 5, the last and smallest group of UPS-related genes responsible for NDD comprises the two E1 ubiquitin-activating enzymes *UBA5* (Duan et al., 2016; Muona et al., 2016) and *UFC1* (Nahorski et al., 2018). Both of these proteins belong to the recently described Ubiquitin-fold modifier 1 (Ufm1)-conjugation system whose biological relevance remains to be fully understood. Because the only E3 Ufm1 ligase identified so far (i.e., UFL1) is recruited at the cytosolic side of the endoplasmic reticulum (ER) membrane (Wu et al., 2010), it is thought that Ufm1 modification is involved in ER protein quality control and/or homeostasis (Adamson et al., 2016). This notion is in line with recent studies showing that proteins involved in these processes such as RPL26 and RPN1 are cellular targets of the (Ufm1)-conjugation pathway (Walczak et al., 2019; Liang et al., 2020). Most importantly, it seems that Ufm1 modification at the ER represses the unfolded protein response (UPR) (Liang et al., 2020), a pathway known to cause sterile inflammation (Ebstein et al., 2019). It is therefore conceivable that Ufm1 loss-of-function might result in sustained overactivation of the UPR and expression of inflammatory markers. This assumption is in agreement with recent reports showing that Ufm1 attenuates inflammation induced by LPS (Li et al., 2017, 2019). Although patients with UBA5 loss-of-function mutations fail to show noticeable symptoms of inflammation, one can again not exclude that these syndromes are devoid of inflammatory process.

**TABLE 5:** NDD-causing genes encoding components of the Umf1-conjugation pathway and associated syndromes.

Gene	OMIM	Syndrome	References	Described regulator of	References
*UBA5*	617132; 617133	DEVELOPMENTAL AND EPILEPTIC ENCEPHALOPATHY 44; SPINOCEREBELLAR ATAXIA, AUTOSOMAL RECESSIVE 24	Duan et al. (2016); Muona et al. (2016)	NF-κB signaling	Li et al. (2017, 2019); Xi et al. (2013)
*UFC1*	618076	NEURODEVELOPMENTAL DISORDER WITH SPASTICITY AND POOR GROWTH	Nahorski et al. (2018)		

*The potential implication of the gene products in the regulation of innate and/or adaptive is indicated. When available, the OMIM (Online Mendelian Inheritance in Man^®^) disorder number is also reported.*

## Conclusion and Future Directions

In this review, we have identified 62 reported monogenic NDD directly caused by lesions in genes encoding components of the UPS (Tables 1–5). To our surprise, 37 of these genes encode products that have been shown to regulate the immune system at various levels (Tables 1–5). Specifically, 20 of them are negative regulators of the two major (i.e., NF-κB and IRF) pathways in inflammation and antiviral response as well as type I IFN signaling (Figure 2). We believe that this number is likely underestimated, as many cellular targets of the identified ubiquitin ligases and/or DUB encompass proteins involved in DNA/RNA processing which may alert the immune system upon dysfunction through the generation of dangers signals. Altogether, this analysis strengthens the straightforward assumption that uncontrolled inflammation contributes to the pathogenesis of psychiatric disorders including NDD, although this remains to be formally demonstrated. One general contradiction stemming from our work is the fact that a great majority of NDD patients does not exhibit typical symptoms of chronic inflammation. However, suspecting subtle and stealthy levels of inflammation remains a challenge for pediatricians and it is likely that children apparently devoid of clinical signs of inflammation are not tested for immune disorders. In this regard, the absence of standardized diagnostic assays for a number of pro-inflammatory cytokines, particularly type I IFN, makes also the detection of specific and atypical inflammatory signatures difficult. One further possible explanation for this discrepancy may be that the UPS components affected in NDD exhibit a tissue-specific distribution, thereby promoting a more localized inflammation rather than a systemic one. In view of the neuronal phenotype of these disorders, it is highly likely that most of these genes are expressed in the CNS including microglia cells and astrocytes which might represent a potential source of inflammation in response to UPS dysfunction. As such, it is conceivable that inflammation might be restricted to the cerebrospinal fluid (CSF) in these patients. Future work aiming to address the role of pro-inflammatory mediators on neuron differentiation and/or function will help improve our understanding of disease pathogenesis and identify therapeutic targets for NDD.

## Author Contributions

FE conceived, wrote, and edited the manuscript. SK, JJP, and EK participated in data analysis and provided intellectual input into the manuscript. FE and SK have designed the figures and tables.

## Conflict of Interest

The authors declare that the research was conducted in the absence of any commercial or financial relationships that could be construed as a potential conflict of interest.

## Publisher’s Note

All claims expressed in this article are solely those of the authors and do not necessarily represent those of their affiliated organizations, or those of the publisher, the editors and the reviewers. Any product that may be evaluated in this article, or claim that may be made by its manufacturer, is not guaranteed or endorsed by the publisher.
